# Identification of tungiasis infection hotspots with a low-cost, high-throughput method for extracting *Tunga penetrans* (Siphonaptera) off-host stages from soil samples–An observational study

**DOI:** 10.1371/journal.pntd.0011601

**Published:** 2024-02-20

**Authors:** Abneel K. Matharu, Paul Ouma, Margaret M. Njoroge, Billy L. Amugune, Ayako Hyuga, Francis Mutebi, Jürgen Krücken, Hermann Feldmeier, Lynne Elson, Ulrike Fillinger

**Affiliations:** 1 Freie Universität Berlin, Institute for Parasitology and Tropical Veterinary Medicine, Berlin, Germany; 2 International Centre of Insect Physiology and Ecology, Human Health Theme, Nairobi, Kenya; 3 Makerere University, College of Veterinary Medicine, Animal Resources and Biosecurity, Kampala, Uganda; 4 Charité–Universitätsmedizin Berlin, Institute of Microbiology, Infectious Diseases and Immunology, Berlin, Germany; 5 KEMRI-Wellcome Trust Research Programme, Centre for Geographic Medicine Research (Coast), Kilifi, Kenya; 6 University of Oxford, Centre for Tropical Medicine and Global Health, Oxford, United Kingdom; University of Maryland School of Medicine, UNITED STATES

## Abstract

**Background:**

The sand flea, *Tunga penetrans*, is the cause of a severely neglected parasitic skin disease (tungiasis) in the tropics and has received little attention from entomologists to understand its transmission ecology. Like all fleas, *T*. *penetrans* has environmental off-host stages presenting a constant source of reinfection. We adapted the Berlese-Tullgren funnel method using heat from light bulbs to extract off-host stages from soil samples to identify the major development sites within rural households in Kenya and Uganda.

**Methods and findings:**

Simple, low-cost units of multiple funnels were designed to allow the extraction of >60 soil samples in parallel. We calibrated the method by investigating the impact of different bulb wattage and extraction time on resulting abundance and quality of off-host stages. A cross-sectional field survey was conducted in 49 tungiasis affected households. A total of 238 soil samples from indoor and outdoor living spaces were collected and extracted. Associations between environmental factors, household member infection status and the presence and abundance of off-host stages in the soil samples were explored using generalized models. The impact of heat (bulb wattage) and time (hours) on the efficiency of extraction was demonstrated and, through a stepwise approach, standard operating conditions defined that consistently resulted in the recovery of 75% (95% CI 63–85%) of all present off-host stages from any given soil sample. To extract off-host stages alive, potentially for consecutive laboratory bioassays, a low wattage (15–25 W) and short extraction time (4 h) will be required. The odds of finding off-host stages in indoor samples were 3.7-fold higher than in outdoor samples (95% CI 1.8–7.7). For every one larva outdoors, four (95% CI 1.3–12.7) larvae were found indoors. We collected 67% of all off-host specimen from indoor sleeping locations and the presence of off-host stages in these locations was strongly associated with an infected person sleeping in the room (OR 10.5 95% CI 3.6–28.4).

**Conclusion:**

The indoor sleeping areas are the transmission hotspots for tungiasis in rural homes in Kenya and Uganda and can be targeted for disease control and prevention measures. The soil extraction methods can be used as a simple tool for monitoring direct impact of such interventions.

## Author summary

The sand flea *(Tunga penetrans*), is the cause of a tropical parasitic skin disease (tungiasis) that is severely neglected by scientists and policy makers yet it affects millions of people in the Americas and Sub-Saharan Africa. The adult female sand flea burrows into the skin, especially in the feet where it grows and expels hundreds of eggs that than develop off-the host on the ground. These environmental off-host stages are responsible for constant re-infection even if a person was medically treated. It was not known up to date where exactly the off-host stages are. Our study provides for the first time direct evidence that indoor floors, specifically those where people with tungiasis sleep, are hotspots for sand flea development and consequently for tungiasis transmission in East Africa. Based on our results, we recommend the interruption of the life cycle and hence prevent re-infection with locally targeted environmental prevention measures such as improving indoor floors with sealed and easily cleanable surfaces or the application of insecticides on indoor floor surfaces.

## Introduction

The sand flea, *Tunga penetrans*, in the order Siphonaptera, is the smallest known flea, with the unfed adult males and females being only 0.8–1 mm in length [1]. The female sand flea penetrates the epidermis of the host, head forward, and embeds itself within 6–8 hours firmly into the skin leaving the rear abdominal segments protruding which allows the flea to breathe, defecate, mate and expel eggs while being constantly attached with its mouth parts to a blood vessel for feeding [1]. Males live freely on the host, blood-feed on the host and mate with the embedded females [2]. Once fertilized, the female undergoes an uncommon hypertrophy, growing 2000-fold to the size of a pea within a week, developing hundreds of eggs which will be expelled over a period of two weeks. After four weeks the embedded parasite starts to shrink and eventually dies. The remains are removed by the processes of skin regeneration. The parasitic skin disease resulting from sand flea infections is known as tungiasis, a highly neglected tropical disease endemic to the Americas, Caribbeans and sub-Saharan Africa. The rapid growth of the female flea causes inflammation with immense pain and itching, secondary infections, and difficulty in walking, and sleeping. Chronic infection with high parasite burden leads to disabilities and significantly reduced quality of life [3]. People with tungiasis are subject to stigma and discrimination [4].

Tungiasis is most prevalent among children under 15 years, the elderly, and the disabled in resource poor communities [5,6]. However, the disease has a very heterogeneous distribution in endemic countries, both spatially as well as temporally [7] which is yet poorly understood, due to the limited amount of research data and absence of national surveillance in affected countries. This is largely attributed to the lack of recognition of this debilitating disease by global and national policy makers and funders [4]. In some communities in Kenya and Uganda, over 60% of households are affected by the disease [5,8], similarly school-based surveys have shown equally high prevalence among primary school pupils in severely affected communities [6]. To date there is no simple and safe method of treatment registered or recommended in endemic communities [9]. Furthermore, treatment alone will not be able to eliminate the disease, given that the causative parasite, like all fleas, undergoes a complete metamorphosis and has an environmental development stage (off-host stages). If not targeted for disease prevention, this environmental stage will always contribute to re-infection. When eggs are expelled from the embedded female and deposited on the floor in a suitable environment, larvae hatch from the eggs 3–4 days after and develop over several larval stages until they pupate 10–14 days after hatching from the eggs. The pupae stage takes another 10–15 days until adults emerge ready to infect the next host [10].

Immature stages of *T*. *penetrans* develop in the upper layer of earthen or sandy surfaces, hence the common name ‘sand flea’ [10]. The biology of the sand flea, specifically its environmental stage, has not been well studied. The limited attention that this parasitic disease received, has been largely in the medical field, to describe case studies or investigate potential treatments [3]. Off-host stages are very small and cannot easily be seen with the naked eyes. The environment where they might be located in the living and activity spaces of infected human and animal hosts is vast and hence significant sampling effort must be invested to investigate and identify the key development sites. Previous descriptions of off-host sampling includes taking small amounts of soil samples from sites where there was a high likelihood of finding specimen, sieving the soil and screening these samples unconcentrated under a stereomicroscope [10–12]. This method is highly inefficient, given it is very time consuming, samples cannot be stored before screening, and individual samples are very small, potentially missing organisms that have a very clustered distribution in the environment [11].

The Berlese-Tullgren funnel method is a well-established entomological tool used for the quantification of microarthropods [13] for example from bird nests, leaf litter, and soil. This tool is well established for extraction of mites and ectoparasitic fleas on animals for ecological and taxonomic studies [14,15]. Aiming at the identification of major *T*. *penetrans* development sites, we opted to modify and calibrate the Berlese-Tullgren funnel method to allow a high-throughput of small soil samples simultaneously, maximising the number of off-host stages resulting from the samples. The method uses a temperature gradient created over the samples in the funnel by the heat emitted from a light bulb which causes the juvenile flea stages as well as any adult fleas that might be present in the soil to burrow down and away from the light and heat source as they fall through a mesh into a collecting container at the bottom of the set-up [15]. This mechanism is based on the natural self-preservation behaviour of the flea larvae since they are negatively phototactic (moving away from light) and positively geotropic (moving downwards) [16].

The objectives of the study were to: (1) assess and optimize the efficiency of extracting off-host stages from soil samples with modified Berlese Tullgren funnels; and (2) identify the major development sites and hence likely disease transmission hotspots for tungiasis in rural areas in Kenya and Uganda, East Africa.

## Methods

### Ethics approval and consent to participate

Protocols for this study were approved by the Kenya Medical Research Institute (KEMRI) Scientific Ethics Review Unit (Non-KEMRI protocol no. 652 and Non-KEMRI protocol no. 644) and by the Makerere University Higher Degrees, Research and Ethics Committee (HDREC protocol number 157) and the Ethikkomission of the Charité Berlin (reference number EA2/100/16) and NACOSTI Research Permit (NACOSTI/P/21/11183). Written consent was obtained from the household heads for soil sampling and household observations. Verbal consent was sought from all household members before any household observations. Standard operational protocols while handling soil samples from the field to the laboratory were followed to prevent any risk of escaping sand fleas.

### Study areas

For the initial design and calibration of the soil extraction process in the laboratory, off-host stages were recovered from soil samples taken from floors of houses with tungiasis infected family members in Msambweni sub-county, coastal Kenya and the experiments implemented at the Muhaka Field Station of the International Centre of Insect Physiology and Ecology (ICIPE) located within the sampling location.

**Fig 1:**
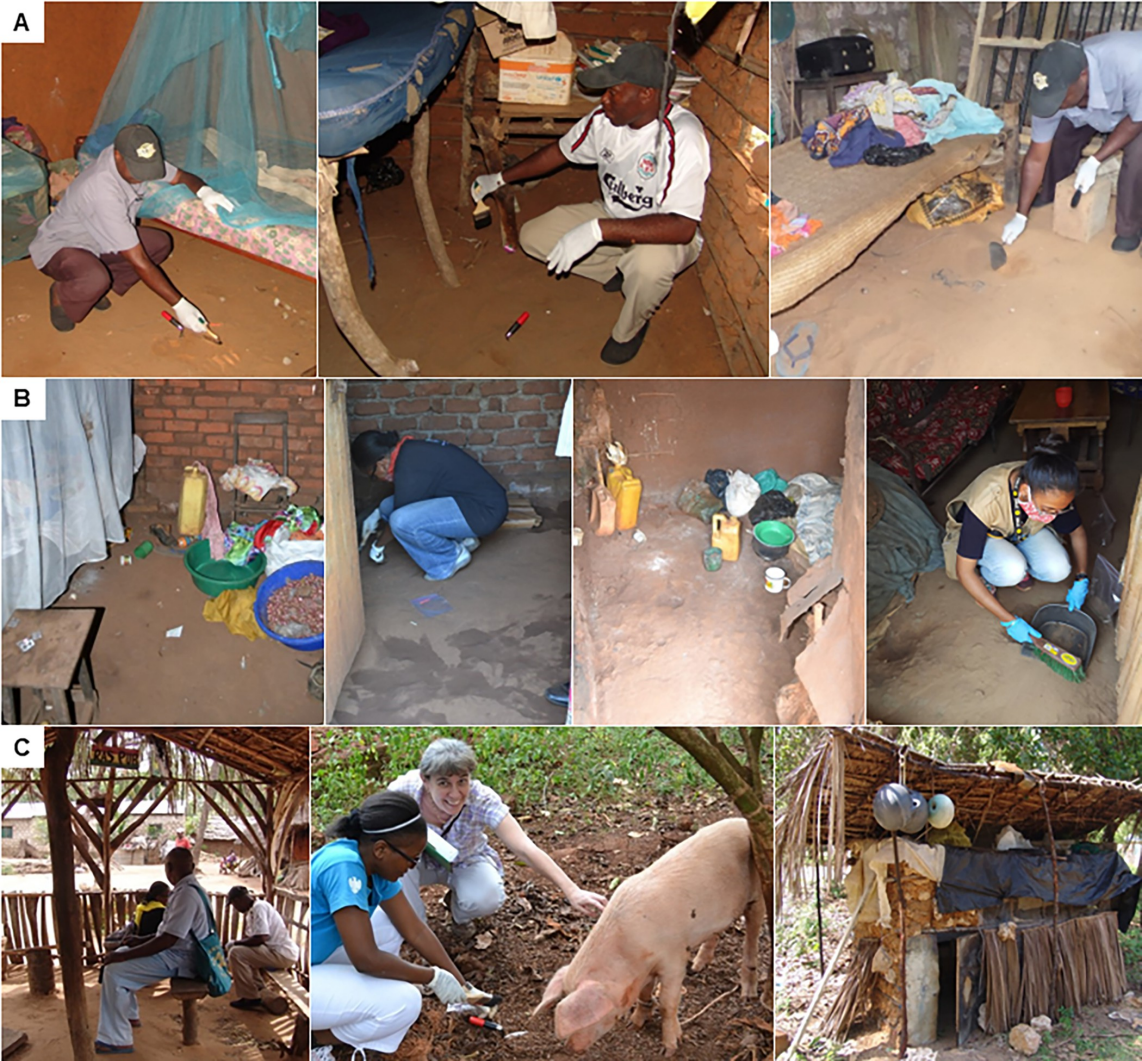
Examples of representative soil sampling areas from different households. Loose sand floors in coastal Kenya **(A)**; compacted clay floors in eastern Uganda **(B)**; and outdoor sitting location (Kenya), animal tethering place (Uganda) and an outdoor kitchen (Kenya) as examples of outdoor sampling locations **(C)**.

To identify the major environmental development sites in the field, cross-sectional surveys were conducted in 19 households in Dabaso Ward, Kilifi North sub-county, coastal Kenya and in 30 households in the south-eastern region of Jinja District, Uganda. These locations were selected based on ongoing work in the areas by the investigators. The number of households chosen for observations and soil sampling was based on feasibility (number of households that could be sampled within three days in the field sites). All households were purposely identified by primary health care workers, who had pre-existing lists of households that were severely affected by tungiasis. The infection status of all household members present during the surveys was established through rapid assessments [17] of the feet and hands implemented by trained primary health care workers. The sleeping place of every household member was recorded. The 19 houses in Kenya were traditional single-room mud houses, with unimproved natural floors largely containing loose sand with a very low clay content as per simple estimation of soil texture by hand (Fig 1; [18]). Out of the 30 houses surveyed in Uganda, 12 were brick-houses with between 2–6 rooms whilst the remaining 18 were traditional mud houses with 1–4 rooms. All houses had unimproved natural floors, but contrary to the Kenyan study locations, floors were compacted and due to the very high clay-content in the local soil which also served for brickmaking, floors were relatively hard with a rough surface (Fig 1).

During the sampling activities, household participants with infections were referred to the nearest health facility where they were treated according to national guidelines [19]. Soil sample extractions were done with portable Berlese-Tullgren extractors directly in the field sites and extracted arthropods preserved in 60% ethylene glycol until morphological identification and quantification in the laboratory.

### Soil sampling

*Tunga penetrans* off-host stages are small and fragile and hide from light between sand, soil and dust grains in the upper 1 cm layer of loose floor materials [10,20]. There they feed, ingesting organic materials with soil particles [21]. We used small hand brooms and dust pans to gently sweep larger, compacted floor surface areas, and various sized paint brushes, between 2 cm and 10 cm wide, to collect dust and debris from cracks and crevices of the floor. Masonry trowels were used to sample floors with loose sand where an upper layer of maximal 1 cm of the soil was collected by carefully inserting the trowel sideways and using a brush to help. In circumstances, where the floor was very hard with very little to no dust at all, the trowels were used to scrape off some material from the floor after initially using hand and paint brushes to collect any loose dust.

For the initial calibration experiments of the extraction method, samples were only taken indoors from areas around the bed of tungiasis infected household members. For the field surveys, more comprehensive sampling was done in indoor and outdoor locations where the family members spent time during the day and night. For compacted clay floors indoors, the entire room floor surface was swept with a hand broom and dust was sampled out of depressions in the floor with brushes. For sampling loose sand floors, we used the trowel and brushes and took small samples from at least 6 locations around the room or outdoor spaces. In bedrooms, special care was taken to sample at various points around the bed or sleeping surfaces on the floor, specifically the foot end. Where people slept on the floor on mats, these were lifted, shaken carefully and samples taken especially from under the foot end. Similarly, any beddings were shaken carefully in front of the bed and any debris originating from them, included in the sample. Outdoor samples were taken from locations where people spent significant amount of time, such as kitchen spaces, eating or resting places and the soil was taken from areas where the feet rested. These outdoor work and resting places where people spent significant time were usually shaded most of the day. We did not sample outdoor places fully exposed to the sun, as it is not plausible that the fragile *Tunga* off-host stages with their very limited mobility would survive extreme heat. The functional use of rooms and spaces sampled was recorded specifically if the room was used for sleeping or not. In the case where houses had several rooms, we also identified all spaces where infected and uninfected household members slept within the household to be able to link soil samples to sleeping areas of infected and non-infected household members.

Soil samples from single rooms or locations were pooled in a plastic ziplock bag (27 x 20 cm) and labelled with a unique record ID for each house, location and sampling event. However, if the sample exceeded approximately 300 g, several bags were used to prevent spilling. Bags were stored in cooler boxes for transport.

### Extraction of *T*. *penetrans* from soil samples with Berlese-Tullgren funnels

**Fig 2:**
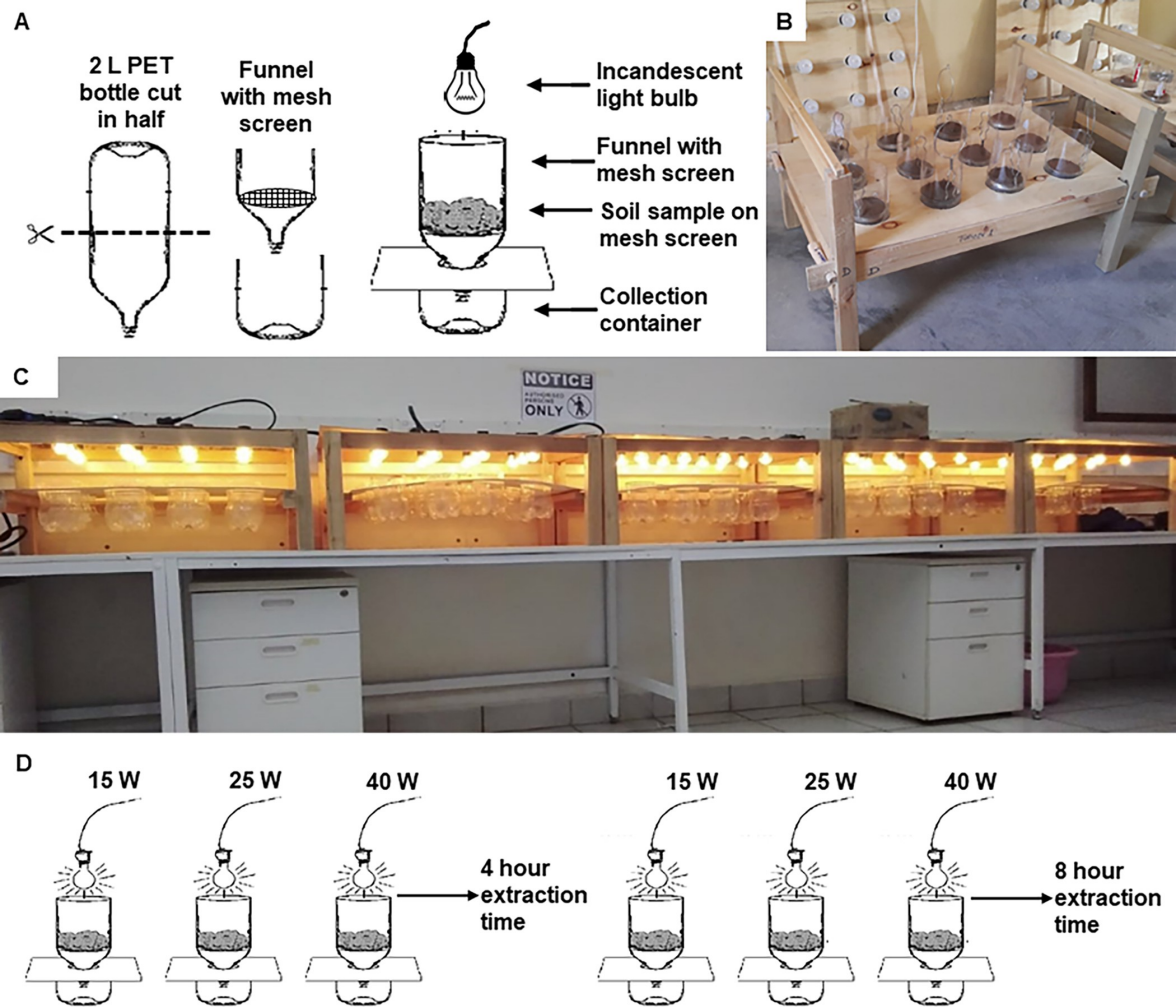
Berlese-Tullgren funnel apparatus for extraction of *Tunga penetrans* off-host stages from soil samples. Schematic diagram showing components of individual funnel made from 2 L PET soda bottle, mesh screen and light bulb **(A)**. Wooden frame with twelve units of Berlese-Tullgren funnels **(B)**. High-throughput set up of 60 Berlese-Tullgren Funnels assembled in 5 units in the laboratory **(C)**. Experimental set-up for the calibration of optimum conditions using 15, 25 and 40 W bulbs and 4 versus 8 hours of extraction **(D)**.

Whilst Berlese-Tullgren funnels are commercially available, they are usually designed for larger samples of leaf litter, rather than small amounts of sand and are relatively expensive. Since at any given sampling date, we were likely to have a large number of samples and our samples required quick extraction preferably within 48 hours after collection to avoid any biohazard from emerging adult fleas, there was need to design the extraction apparatus for high throughput (Fig 2). The individual extraction funnels were made from used 2 L polyethylene terephthalate (PET) soda bottles, which were thoroughly washed and cut in half, the upper half inverted, serving as the funnel and the lower half as the collection container (Fig 2). We used stainless steel fine mesh strainers with a diameter of 7.5 cm (diagonal measurement of holes 0.7 mm) as mesh screen for placing the soil/sand sample on top. For this, we cut off the handle and inserted the strainer into the plastic funnel ensuring a tight fit. A wooden stand was fabricated with holes cut to hold 12 extraction funnels and their associated collection containers. Incandescent light bulbs were used as light and heat sources. Arthropods which are heat and light sensitive descend downward away from the heat, through the mesh and into the collection container. The bulbs were suspended over the samples with the help of a plywood board through which they were held in position on top of each funnel 10.5 cm above the soil sample. The bulbs were connected at every 15 cm distance with E27 bulb sockets with silicone gaskets to a heavy-duty outdoor (IP44) illumination cord set (ILLU, Germany) consisting of a flat cable (H05RNH2-F2x1,5; 5 mm x 13 mm) with plug and end piece. Five units enabled us to extract 60 soil samples at the same time. The mobility of these units also allows extraction of samples directly in the field as long as there is a constant supply of electricity.

### Experiments to calibrate the Berlese-Tullgren funnel extraction

To assess how efficient the extraction process is in quantifying the number of larvae present in any given sample, and to develop settings to maximise this efficiency, we implemented experiments using three different bulb wattages (15 W, 25 W and 40 W) and two extraction periods (4 hours and 8 hours) (Fig 2). To be able to collect alive off-host larvae stages, the collection container was kept empty (not containing a killing and preservation agent).

We spiked a constant amount of 100 g of soil with 10 alive flea larvae of similar size. This soil was collected from houses without infected family members, to ensure it was similar to the soil we usually find *T*. *penetrans* larvae in. We exposed the soil to the sun and then sterilised it in batches of 100 g in a pressure cooker for 1 hour. This was done to ensure that there were no other alive organisms in the soil that might interfere with the results. The larvae were obtained from soil samples collected from houses with tungiasis infected household members. These larvae were extracted alive within 24 hours of the experiment by exposing the collected soil to heat from 25-Watt bulbs over an extraction time of 8 hours. The spiked soil was then placed on top of the metal sieves before switching on the light bulbs. The number of alive and dead larvae extracted in the collection container were recorded and then transferred for storage in 70% ethanol. Each of the six treatment combinations (Fig 2) was replicated three times on different days with fresh batches of larvae. The temperatures of the soil samples were measured just a few mm below the top at the end of the extraction time using a thermometer (Armored Thermometer, Lamotte Company, USA).

### Extraction of field samples

Every soil sample from the field was weighed in its plastic ziplock bag prior to placing in the Berlese-Tullgren funnels. After weighing, the bags were placed in a -20° C freezer for 10–15 minutes to immobilise but not kill any adult fleas or any other arthropods in the soil sample that might escape during the transfer from the bag into the funnel. Each extraction funnel was labelled according to the information on the bag and received a maximum of 100 g of soil/sand. If samples were larger, they were split and extracted with several funnels as applicable. The resulting arthropods from the collection chambers were in the end pooled again for samples from the same collection site. Once the samples were filled in, the cable was plugged into the electricity. Samples from the field surveys were exposed to heat from a 40-Watt light source overnight for 12 hours. Samples from the field needed immediate killing and preservation of the extracted arthropods, hence the collection container was filled with 50 ml of 60% ethylene glycol. Once the extraction was stopped, the soil samples were returned to their respective plastic ziplock bags, transported to the laboratory and discarded as biohazard waste. The contents of the collection containers with ethylene glycol were poured into well labelled petri dishes where they were allowed to settle overnight. Flea off-host stages were identified using a Zeiss Stemi 508 stereomicroscope (magnification 6.3x).

### Flea identification

**Fig 3:**
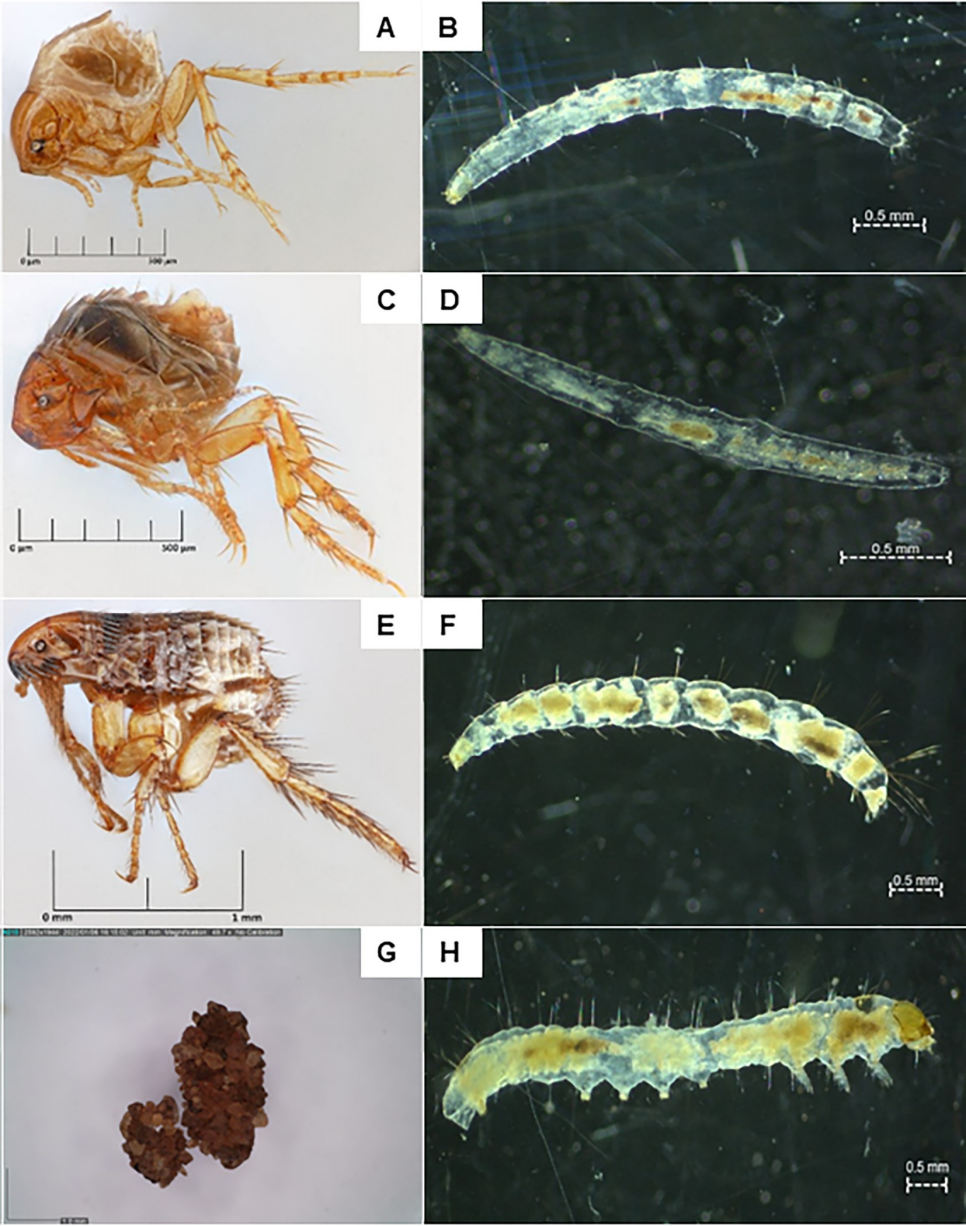
*Tunga penetrans* (sand flea) adult **(A)** and larva **(B)**; *Echidnophaga gallinacea* (stick tight (hen) flea) adult **(C)** and larva **(D)**; *Ctenocephalides felis* (cat flea) adult **(E)** and larva **(F)**; Pupa cocoons of *Tunga penetrans* (small on left) and *Ctenophalides felis* **(G)**; Polypod insect larva [24] which can be distinguished from the flea larvae by the 3 pairs of legs and 5 pairs of prolegs (Order Lepidoptera) **(H)**. Photos of adult fleas captured using Macropod Pro macrophotography system (Macroscopic Solutions.com), photos of larvae captured using Axiocam ERc 5s Rev, 2.0 mounted on the Zeiss Stemi 508 stereo microscope (Carl Zeiss Suzhou Co., Ltd.) and photos of pupa cocoons captured using a DinoCapture 2.0 (Dunwell Tech., Inc. Dino-Lite US).

Whilst adult flea species have been well described with morphological identification keys available [22], there is very little information on the taxonomic features of flea larvae in general [23] and no identification key available to distinguish between the immature stages of different flea species. We describe our identification methods in more detail in the below section in an attempt to provide a first tool to identify *T*. *penetrans* off-host stages. We recently developed a molecular identification tool to distinguish *T*. *penetrans* larvae from other flea species’ larvae in soil samples [21], which enabled us to confirm our identifications and establish a set of morphological differences between *T*. *penetrans* larvae and other common flea species’ larvae frequently encountered in our sampling sites (Fig 3).

Flea larvae are small, depending on the species, anything between 1.5–10 mm, have a wormlike body of 13 segments (3 thoracal and 10 abdominal) without any legs, have a firmly sclerotised head capsule with mandibles but without compound eyes. Two hooks, called anal struts, project backwards and downwards from a swollen anal mound on the last (10^th^) segment. The larval body is translucent white with a darker coloured gut that can be seen through the skin. Before pupating, the larval body somewhat shortens in length which gives them a short, fatter look. These late-stage larvae spin a cocoon which attracts debris and sand in which they pupate and develop into the adult flea (Fig 3 [23];).

In our soil collections, we encountered off-host stages of three flea species. The species were confirmed by separating what appeared three distinctly different morphological types of larvae and letting them emerge into adults. *Tunga penetrans* larvae were further confirmed using molecular tools [21]. The three species were *T*. *penetrans* (sand flea), *Ctenocephalides felis* (cat flea) and *Echidnophaga gallinacea* (stick tight or hen flea). The major morphological characteristics for the differentiation of species are the setae (bristles) that cover the head capsule as well as the body segments. Observations were made in regard to the morphological similarities and differences of the three larval species that might guide field teams.

The stick tight flea (*E*. *gallinacea*) larvae were the only ones that might be easily mistaken for *T*. *penetrans*. The stick tight flea larvae were smallest, with an average body length of 2.7 mm (ranging from 2.1 mm to 3.2 mm). Its most striking feature was the absence of any setae in the largest possible magnification using a Zeiss Stemi 508 stereo microscope. This contrasts with *T*. *penetrans*, which was found to be slightly longer on average (3.5 mm, ranging from 2.7 mm to 4.1 mm), and showcasing short setae on all body segments. These setae are of consistent length on all abdominal segments (Fig 3 and Table 1). Both, the stick tight flea and the sand flea larvae can be easily distinguished from cat flea larvae and other representatives of the Ceratophyllidae, by their body lengths and by the bristly-haired abdominal segments, with increasing lengths towards abdominal segments 9 and 10 (Fig 3 and Table 1).

**Table 1:** Morphological features of the larvae of *Tunga penetrans*, *Echidnophaga gallinacea*, and *Ctenocephalides felis*.

Species	n	Mean measurements in millimetres ^a^ with (95% confidence interval) and {range}				
		Body length	Setae length on head segment	Setae length on thoracic segments 2–3	Setae length on abdominal segments 4–9	Setae length on abdominal segment 10
*Tunga penetrans*	10	3.5 (3.2–3.8) {2.7–4.1}	0.05 (0.05–0.06) {0.04–0.07}	0.08 (0.07–0.09) {0.06–0.09}	0.08 (0.07–0.09) {0.07–0.1}	0.1 (0.08–0.1) {0.08–0.2}
*Echidnophaga gallinacea*	8	2.7 (2.3–3.1) {2.1–3.2}	-	-	-	-
*Ctenocephalides felis*	10	4.5 (4.2–4.8) {3.9–5.2}	0.07 (0.06–0.08) {0.04–0.1}	0.1 (0.09–0.1) {0.08–0.1}	0.2 (0.2–0.3) {0.2–0.3}	0.5 (0.5–0.6) {0.4–0.6}

^a^Larvae were preserved in 70% ethanol at the time of measurement which likely led to increased body length compared to alive specimen [25].

### Statistical analyses

All analyses were carried out using R statistical software (R-4.2.1) or IBM SPSS Statistics v.29.0.0.0. Proportions of larvae successfully extracted (dead and/or alive) during the calibration experiments were analysed using generalised linear models fitted with quasibinomial data distribution and a logit link function generating odds ratios (OR) with their associated confidence intervals (CI). Bulb wattage and the extraction time were included in the model as explanatory variables. The denominator for the Berlese-Tullgren Funnel extraction experiment was the total number of larvae introduced per round. All mean percentages and their 95% confidence intervals (CI) reported in the result section were estimated based on the model by transforming the log odds (logit) of the outcome to the odds scale and from the odds scale to the probability scale.

Generalized estimating equations were used to analyse the data from the field survey. The household identification numbers were included as repeated measures. Proportions were analysed with a binary logistic model generating ORs, whilst counts were analysed by fitting a negative binomial distribution with log link function generating rate ratios (RRs) and their associated confidence intervals. All reported means (proportions and counts) and their 95% CI were estimated based on the model. Data from Kenya and Uganda were initially explored separately and with models where interactions between country and other explanatory variables were tested. Since the trends for all explored outcomes were similar for samples from both countries and no significant interactions were found, we combined the data from both countries for the final models presented in the results.

## Results

### Establishing optimal conditions for extraction of flea off-host stages from soil samples

**Fig 4:**
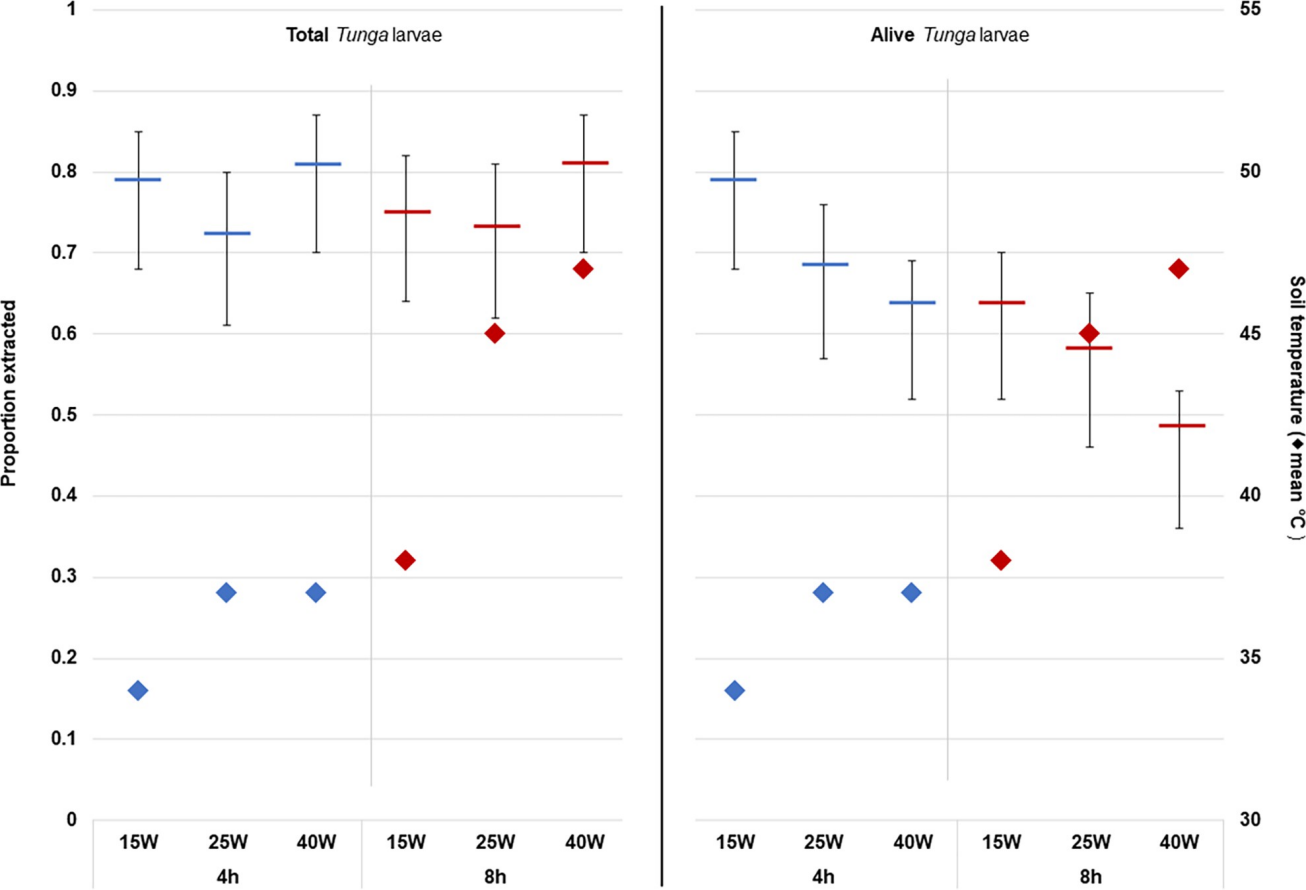
Model estimated mean proportions (including 95% confidence intervals) of larvae extracted (total versus alive) from spiked soil samples in relation to bulb wattage and extraction time. Mean soil temperature is shown on secondary axis.

The experiments revealed that the recovery of combined dead and alive larvae in collection containers, can be expected to be around 75% (95% CI 63–85%) of the larvae contained in the soil, irrespective of the wattage of the heat source or time for extraction (Fig 4). The yield of alive larvae, however, was significantly, and independently associated with both, the wattage of the heat source and the time of extraction (Table 2 and Fig 4).

**Table 2:** Outputs from generalised models exploring the association between the proportion of *Tunga* larvae recovered and bulb wattage and extraction time in experiments.

	Odds ratio (OR) (95% CI)	*p-*value
**Total (dead and alive) larvae**		
**Bulb energy**		
15W	1	
25W	0.79 (0.50–1.26)	0.325
40W	1.26 (0.77–2.03)	0.360
**Duration**		
4h	1	
8h	0.96 (0.66–1.40)	0.835
**Alive larvae**		
**Bulb energy**		
15W	1	
25W	0.67 (0.40–1.11)	0.123
40W	0.49 (0.30–0.81)	0.008
**Duration**		
4h	1	
8h	0.53 (0.36–0.80)	0.003

Using a 40-Watt bulb reduced the odds of finding a larva alive by 51% and using a 25-Watt bulb reduced the odds by 47% as compared to extracting larva with the help of a 15-Watt bulb. Similarly, exposing the soil sample to eight hours of heat reduced the odds of finding alive larva in the collection container by 47% as compared to a four-hour regimen (Table 2). As expected, the wattage of light bulbs used was closely correlated with the temperature in the soil with median temperatures being over 10 degrees Celsius higher when the 40-Watt bulb was used for 8 hours, than when shorter exposure and lower bulb-wattage was used (Fig 4).

### Identification of major environmental development sites of *Tunga penetrans*

From the 49 households that were surveyed, a total of 237 soil samples were taken, 167 from indoor and 70 from outdoor locations, and placed in the Berlese-Tullgren funnels for extraction of off-host stages. The mean weight of individual soil samples was 156 g (95% CI 138–168 g). We were able to confirm the presence of *T*. *penetrans* off-host stages from at least one soil sample from 43 out of the 49 households (88% of all households sampled). Out of all the 237 samples analysed, *T*. *penetrans* off-host stages were recovered from 97 samples (41%). The average number of larvae extracted from these positive samples was 9.3 (95% CI 5.1–17.2).

Further analysis revealed a very strong association of the presence of off-host stages with the indoor sampling location. The odds of a soil sample being positive was 3.7 times higher when it was collected indoors than when it was collected outdoors. On average, 49% of all indoor samples contained off-host stages, whilst only 21% of the outdoor samples contained off-host stages (Table 3).

**Table 3:** Bivariate analyses results investigating the prevalence and abundance of *Tunga* off-host stages in relation to sample location.

Prevalence of off-host stages			
	Mean* percentage (95% CI)	Odds Ratio (OR) (95% CI)	*p*-value
**Sampling location**			
Outdoors	21 (12–33)	1	
Indoors	49 (43–55)	3.7 (1.8–7.7)	<0.001
**Use of indoor location**			
Not sleeping	23 (8–52)	1	
Sleeping	53 (46–61)	3.7 (1.1–13.5)	0.045
**Tungiasis infection status of resident**			
without tungiasis patient	16 (8–31)	1	
with tungiasis patient	67 (57–76)	10.5 (3.9–28.4)	<0.001
**Abundance of off-host stages**			
	Mean* **counts (95% CI)**	**Rate Ratio (RR) (95% CI)**	***p*-value**
**Sampling location**			
Outdoors	1 (4–2.8)	1	
Indoors	4.3 (2.4–6.8)	4.1 (1.3–12.7)	0.014
**Tungiasis infection status of resident**			
without tungiasis patient	0.5 (0.2–1.1)	1	
with tungiasis patient	6.4 (4.1–10.2)	13.8 (5.3–36.1)	<0.001

*based on model parameter estimates

In the indoor environment, samples were taken from sleeping locations as well as from other locations where household members might spend some time, but where no one slept at night. The odds of the presence of off-host stages were 3.7 times higher from samples around sleeping areas than from samples taken in indoor locations not used for sleeping (Table 3). When considering the infection status of household members in the analysis, the associations became even stronger. Finding a soil sample positive for sand fleas was 10.5 times more likely when the sample was taken from a room where an infected member slept than when taken from a room without an infected member sleeping at night. Of the samples taken from sleeping locations with tungiasis patients, 67% were positive, whilst only 16% of the samples taken from indoor locations without a patient were positive (Table 3).

The same associations as observed for the prevalence were also observed for larval abundance. For every one larva extracted from an outdoor sample, there were 4 larvae extracted from an indoor sample. Indoors, we see that for every larva sampled in a room where no infected household member slept, we recovered 14 larvae from rooms with an infected household member sleeping (Table 3). Notably, the abundance of off-host stages in samples was positively associated with the number of infected people sleeping in the room (RR 1.84 95% CI 1.50–2.27).

Livestock keeping was rare amongst the households that were sampled, and hence we could not evaluate the risk associated with animal husbandry practices on outdoor occurrence and abundance of off-host stages. We were only able to take four soil samples in Uganda from places where households had tethered a pig which was not allowed to roam freely. All four samples were positive for off-host stages.

## Discussion

Our study confirmed that indoor sleeping areas are the major environmental development sites for *T*. *penetrans* in rural Kenya and Uganda and hence the sites where infection and re-infection takes place. Specifically, the data showed that off-host stages can be found in close association with the sleeping areas of tungiasis infected household members and their abundance increases as the number of infected persons increases. Our study is the first to provide direct proof of off-host development sites for *T*. *penetrans* in sub-Saharan Africa. The observation is in line with data published from Brazil [11] where off-host stages of *T*. *penetrans* were also closely associated with people’s or animals’ sleeping locations, which in the investigated Brazilian communities might have been inside proper houses or in more makeshift temporary shelters.

Interestingly, in multi-roomed households with tungiasis infected household members, not everyone was infected, possibly due to the heterogeneity in the spatial distribution of off-host stages in the house putting people at different risk. In the East African context, cultural norms in some rural communities lead to school aged children sleeping in a separate location from that of other family members and taking care of their own room and hygiene [26]. Since fleas in general are particularly associated with animals that make nests or have other places that they habitually occupy [27], it appears plausible that *T*. *penetrans* also completes its life cycle in close vicinity of its hosts sleeping location where it leads to immediate re-infection. Host seeking cues such as carbon dioxide, host scent, heat and vibration have been suggested as general cues for orientation towards hosts for other flea species [27]. Further studies on the potential host cues used by *T*. *penetrans* to locate the preferred locations around the feet of the host as well as the further investigations on circadian rhythms for egg-laying, hatching of pupae and host-seeking might elucidate the evolutionary adaptation of this parasite to its hosts’ behaviours. It has been shown for other flea species, that host-seeking activity peaks during the hours of the scotophase (dark phase), hence coinciding with the sleeping hours of hosts, whilst in the absence of a host or other stimulations they remain relatively immobile [28]. These rhythms and behaviours likely contribute to maximise the transmission of tungiasis.

Experimental investigations into such behaviours could lead to novel surveillance and control tools, such as attract and kill traps. The ability to collect and extract large numbers of alive off-host stages from soil samples was a first necessary step to enable such experiments in future. The modified Berlese-Tullgren funnels were highly efficient in extracting *T*. *penetrans* off-host stages, resulting in direct evidence of infestation in close to 90% of all households sampled and yielding of around 75% of all off-host stages present in samples. The high throughput setup benefited the quick extraction and storage of samples directly in the field and makes the approach a robust tool for monitoring environmental stages as an estimate of impact of tungiasis control interventions. The calibration experiments showed that a four-hour heat exposure period is sufficient to extract the majority of off-host stages from the samples as long as they are exposed in relatively small volumes of around 100 g. When combined with gentle heat provided by a 15-Watt incandescent light bulb, a high number of larvae can be harvested alive which will aid the implementation of bioassays for example to test the efficacy of insecticides for the control of *T*. *penetrans* off-host stages when applied on floors. Experiments could also further elucidate the development requirements of off-hosts stages such as soil type, organic matter, pH, temperature, or humidity, which in the past have led to a lot of speculations in the available literature [29] aiming at explaining the heterogeneous distribution of the disease [30]. A current limitation for the manufacturing of the extractor is the availability of incandescent light bulbs in general, as they are increasingly being replaced by non-heat emitting LEDs, and of the low-wattage incandescent bulbs specifically in the East African context. Whilst simply monitoring the presence and abundance of off-host stages, a 40-Watt bulb, would be sufficient, and is still widely available in Kenya and Uganda, the extraction of alive off-host stages might require further experimentation with a 40-Watt bulb at a higher distance to the soil to equally reduce the soil temperature and increase the chances of larval survival as they move towards the bottom of the collection container.

The reduced survival of off-host stages in the upper soil layer at high temperatures might also explain why outdoor samples rarely yielded any. Flea larvae are soft bodied without a strong exocuticle to protect them from desiccation, hence, fleas occur in protected microhabitats with suitable conditions where populations can rapidly increase [27]. Our sample size was small, and restricted to two locations that were relatively similar in their environmental conditions, and houses were purposely selected, hence we recommend further studies, preferably employing a random sampling strategy, in different cultural contexts and climates to complete our understanding of the disease ecology. In the present study, we observed that if outdoor development of *T*. *penetrans* exists, it might be in most cases associated with a suitable animal host, especially pigs [31]. Here, however, we did not systematically sample animal resting places, and only had a few leaf litter debris samples taken from pig resting sites, which all were positive. Tungiasis has been shown to be a severe impediment to pig farmers in Nigeria and eastern Uganda [32,33] but reports lack from Kenya. There is need for a more systematic survey on the role of animal hosts in different disease endemic regions and the microhabitat conditions necessary for *Tunga* off-host development when animals are kept in the outdoors.

### Conclusions

Indoor floors, specifically those where people sleep are hotspots for *T*. *penetrans* development and consequently for tungiasis transmission. Our study provides direct evidence in support of previous qualitative risk factor surveys that have associated the unimproved soil floors in traditional rural houses with severe tungiasis infection in East Africa [5]. This finding suggests that the interruption of the life cycle and hence prevention of re-infection is possible by combining tungiasis treatment efforts with environmental prevention measures such as improving indoor floors to provide sealed and easily cleanable surfaces. The application of insecticides on indoor flooring such as insect growth regulators which are often used for the control of immature flea stages in veterinary products [34] might provide an alternative when floor improvement is not an option. The role of animal resting sites requires further investigation as potential development sites for off-hosts stages. The developed high throughput Berlese-Tullgren extraction method can support novel investigations into the *T*. *penetrans* ecology including factors affecting disease seasonality and can serve as a simple monitoring tool for direct intervention impacts.

## Supporting information

S1 Data Database-field soil sampling. (XLSX)

S2 Data Database-optimisation conditions for extraction of off-host stages. (XLSX)
